# Unraveling the Hygiene Hypothesis of helminthes and autoimmunity: origins, pathophysiology, and clinical applications

**DOI:** 10.1186/s12916-015-0306-7

**Published:** 2015-04-13

**Authors:** Mathilde Versini, Pierre-Yves Jeandel, Tomer Bashi, Giorgia Bizzaro, Miri Blank, Yehuda Shoenfeld

**Affiliations:** 1 grid.413795.d0000000121072845The Zabludowicz Center for Autoimmune Diseases, Chaim Sheba Medical Center, Tel-Hashomer, 52621 Israel; 2 Department of Internal Medicine, Archet-1 Hospital, University of Nice-Sophia-Antipolis, 151 Route de Saint Antoine de Ginestière, 06202 Nice, France; 3 grid.12136.370000000419370546The Laura Schwarz-Kipp Chair for Research of Autoimmune Diseases, Sackler Faculty of Medicine, Tel-Aviv University, Tel-Aviv, Israel

**Keywords:** Allergy, Autoantibodies, Autoimmunity, Autoimmune disease, Helminthes, Hygiene hypothesis, Infection, Parasite, T-regulatory cells, Th1

## Abstract

**Background:**

The Hygiene Hypothesis (HH) attributes the dramatic increase in autoimmune and allergic diseases observed in recent decades in Western countries to the reduced exposure to diverse immunoregulatory infectious agents. This theory has since largely been supported by strong epidemiological and experimental evidence.

**Discussion:**

The analysis of these data along with the evolution of the Western world’s microbiome enable us to obtain greater insight into microorganisms involved in the HH, as well as their regulatory mechanisms on the immune system. Helminthes and their derivatives were shown to have a protective role. Helminthes’ broad immunomodulatory properties have already begun to be exploited in clinical trials of autoimmune diseases, including inflammatory bowel disease, multiple sclerosis, rheumatoid arthritis, and type-1 diabetes.

**Summary:**

In this review, we will dissect the microbial actors thought to be involved in the HH as well as their immunomodulatory mechanisms as emphasized by experimental studies, with a particular attention on parasites. Thereafter, we will review the early clinical trials using helminthes’ derivatives focusing on autoimmune diseases.

## Background

For several decades, Western countries have been facing an increasing incidence of allergic and autoimmune disorders [1-6]. Thus, in the United States (US), the prevalence of asthma in children has increased by 38% between 1980 and 2003. Similarly, this rise has reached 56% and 59% between 1964 and 1990 in Scottish and Australian children, respectively [1]. Regarding autoimmune diseases, the incidence of inflammatory bowel disease (IBD) has risen in the past 50 years up to 8 to 14/100,000 persons for ulcerative colitis (UC) and 6 to 15/100,000 persons for Crohn’s disease (CD) [4]. Likewise, the annual increase in incidence of type-1 diabetes (T1D) ranged from 2.9% to 5.4% per year according to the countries during the 1989–2003 period [3]. Also, the number of people living with multiple sclerosis (MS) worldwide has climbed from 2.1 million in 2008 to 2.3 million in 2013 [6]. Quoted here are only few examples illustrating the major public health problem posed nowadays by this epidemic.

Immune-mediated conditions are thought to result from a complex interplay between genetic predisposition, immune dysregulation, and environmental factors [7,8]. Since genetic basis has not undergone any major changes in such a short period of time, environmental factors are highly suspected to be responsible for this recent outbreak. Especially, vitamin D deficiency [9], tobacco [10], air pollution [11], adjuvants [12], and obesity [13] have been incriminated in the pathogenesis and the recent rise in chronic inflammatory disorders. Together with these factors, infections are widely demonstrated to play a critical role in autoimmunity [14]. The so called Hygiene Hypothesis (HH) postulates that the reduced exposure to microorganisms in industrialized countries resulting from improved sanitary conditions would increase immune reactivity, thus promoting the development of allergic and autoimmune diseases [15]. From its first formulation in 1989 [16], this theory has been strengthened by solid epidemiological, experimental, and clinical data, paving the way for future therapies.

In the first part of this opinion piece we discuss epidemiological data, originally from allergic diseases followed by autoimmune diseases, which have given birth and reinforced the concept of the HH. Since these abundant epidemiological observations, numerous experimental studies have clarified the type of microorganisms involved in this theory, thereby specifying their immunoregulatory effects. Thus, a wide spectrum of viruses, bacteria – especially from the gut microbiome – and parasites have been implicated as major actors in this theory. Therefore, there is now a growing and exciting literature regarding the key role played by these microbes in both allergic and autoimmune disorders [17-20].

Herein, we have chosen to focus on the emerging role of parasites, particularly helminthes in autoimmune diseases. First, we will specify their immunoregulatory effects in autoimmune diseases identified through experimental models. Then, we will detail the experimental studies from animal models and the first clinical trials conducted in humans that have resulted from the HH.

### Birth of the Hygiene Hypothesis (HH)

The ability of infectious agents to modulate the immune system has long been a fascinating topic. For almost half a century, infections have been widely demonstrated to act as triggering factors of autoimmune response. Viral agents, such as Epstein-Barr virus, cytomegalovirus, and parvovirus B19, as well as numerous bacteria and parasites have been associated with the presence of a wide variety of autoantibodies and found to contribute to the pathogenesis of autoimmune diseases [14,21]. Thus, parasitic infections have been shown to promote autoimmunity through various mechanisms, including molecular mimicry of parasitic epitopes, alteration of host antigens, polyclonal activation, and expansion of autoreactive B-cell clones, as well as manipulation of the idiotypic network [22].

At the same time, it became increasingly evident that infectious microorganisms could also exert an immunomodulatory and immunodepressant action on the immune system, resulting in a protective effect against immune-mediated conditions such as allergies and autoimmune diseases. The changes that have accompanied the recent modernization in Western countries, such as migration from rural to urban areas, improved sanitation, access to clean water, control of food production, or even vaccination campaigns, have reduced contact with these ancestral microorganisms with which mammals had coexisted and co-evolved for millennia [23]. Hence, the HH concept emerged, postulating that the slightest exposure to these immunoregulatory infectious agents – called ‘Old Friends’ by Rook [24] – in industrialized countries due to improvement in hygiene conditions promotes the development of chronic inflammatory disorders and contributes to their recent rise [15].

This theory was first suggested by Greenwood [25], nearly half a century ago, when reporting a lower prevalence of autoimmune diseases in Nigerians. He suggested an immunomodulatory effect of multiple parasitic infections since childhood, later confirmed by demonstrating that infection of different strains of mice prone to developing autoimmune diseases with the rodent malaria parasite *Plasmodium berghei* prevented the occurrence of the disease [26]. The inverse correlation between the dramatic decrease in infections in industrialized countries due to better hygiene and the concomitant increase in immune-mediated diseases was finally clarified by Strachan in 1989 [16]. Indeed, by following a cohort of more than 17,000 children born in 1958 for 23 years, he observed an inverse relationship between the number of older siblings in the household and the prevalence of hay fever, therefore concluding that allergies could be prevented by infections in early childhood. According to Strachan, a lower exposure to these infections might promote atopic diseases. These observations have led to the birth of a new paradigm on the role of infectious agents in immune disorders. Since then, the HH has been widely powered by epidemiological, experimental, and clinical data.

### Epidemiological evidence

The HH, as formulated by Strachan a quarter-century ago [16], originally focused on allergic diseases. It claimed that their recent rise in Western countries was promoted by reduced exposure to microorganisms due to improved hygiene conditions. Since these early observations, many epidemiological data have reinforced this theory, first on allergic disorders and then extending to autoimmune diseases.

A number of studies have investigated the prevalence of allergic diseases according to living conditions. First, the initial observation of Strachan [16], demonstrating an inverse correlation between the sibship size and the subsequent risk of allergy, has since been widely replicated in a large number of studies in affluent countries [27-30]. Moreover, Strachan et al. [31] recently confirmed, in a broad international study involving more than 500,000 children in 52 countries, the inverse association between the risk of developing hay fever or eczema and the total number of siblings; the association being stronger in more affluent countries. Otherwise, pet ownership has also been linked to a decreased prevalence of allergic diseases. In a recent meta-analysis including 36 publications, Pelucchi et al. [32] reported a favorable effect of exposure to pets, especially to dogs, on the risk of atopic dermatitis in infants or children. Similarly, worst living standards in Eastern Germany compared with Western Germany were associated with a reduced occurrence of atopic diseases [33]. Thereafter, the prevalence of atopy experienced an increase in children in Eastern Germany born after the reunification of Germany in 1990 [34]. Equally, other lifestyle factors, including low antibiotic consumption [35,36] and growing up in rural areas, were associated with a diminished prevalence of allergic diseases [37,38]. In developing countries, an inverse relationship was also observed between the prevalence of parasitic infections, especially helminthic, and the risk of allergic diseases. For example, in Ecuador [39], Gabon [40], and Brazil [41], helminth infections were shown to have a protective effect on allergic reactivity. Conversely, anti-helminthic treatment of chronically infected children in Gabon [40], Venezuela [42], and Vietnam [43] resulted in increased atopic reactivity.

The HH was later extended when the protective effect of infectious agents, especially parasites, against autoimmune diseases was suggested through various epidemiological studies [15]. As previously reported, the number of siblings has been shown to correlate inversely to the risk of MS [44,45]. Furthermore, in the Italian island of Sardinia, several epidemiological and immunogenetic evidences [46-49] link malaria eradication 50 years ago with the concomitant increase of MS. It is assumed that the strong genetic selective pressure of malaria along the centuries led to the selection of polymorphisms and genotypes conferring resistance to *Plasmodium falciparum*, the causative agent of malaria. These polymorphisms are responsible for the increased immune reactivity required to control malaria. As soon as the immunoregulatory organism was withdrawn from the environment by the modern lifestyle, these genetic variants led to excessive inflammation and became susceptibility factors for chronic inflammatory disorders, especially MS.

The Karelia region is also of great interest to investigate the influence of lifestyle and infections on the risk of immune-mediated disorders. This region is divided into a Finnish area, characterized by high standards of hygiene, and a Russian area, with poorer hygiene and an increased rate of infections. Finnish Karelian maintain one of the highest prevalence of autoimmune and allergic diseases, while Russian Karelian prevalence is far lower, despite sharing the same genetic background. For example, the incidence of T1D is six-fold higher in Finland compared to the adjacent Karelian republic of Russia, the wide gap in infection rates between the two regions being strongly suspected to contribute to this difference [50,51]. Subsequently, Weinstock et al. [52] expanded the HH to IBD based on the increasing prevalence of IBD in the US contrasting with the declining prevalence of helminthes. This observation has since been confirmed in other parts of the world. In sub-Saharan Africa, where helminthic infestation is frequent, a low incidence and prevalence of IBD is observed which cannot be explained by genetic factors due to the fact that, in black populations of the US and UK, the incidence of IBD is approaching that of the white populations [53]. Moreover, migration studies have shown that descendants of immigrants coming from a country with a low incidence acquire the same incidence as the host country, as illustrated for T1D [54,55] and MS [56,57]. Similarly, the prevalence of systemic lupus erythematosus (SLE) was found to be much higher in African Americans compared to West Africans [58]. Interestingly, a case–control study in India showed that none of the patients with rheumatoid arthritis (RA) were positive for circulating filarial antigen in contrast to 40% of healthy controls [59].

Such epidemiological observations raise questions concerning the nature of the protective infectious agents involved and the mechanisms through which they modulate the immune system and thus the risk of inflammatory disorders.

These epidemiological data, in conjunction with other data, reinforced the paradigm that infectious agents may confer a protective effect against chronic inflammatory diseases. Therefore, it is of interest to unravel and clarify the mechanisms of this theory.

### Pathophysiology of the Hygiene Hypothesis (HH)

#### Who are the actors in the HH?

The term ‘Hygiene’ in the HH refers to all changes in our lifestyle corresponding to ‘Westernization’ and resulting in greater hygiene levels. These developments have affected, in particular, our living space, food preparation, access to clean water, and medical and therapeutic care, and have thereby led to profound changes in our microbial environment. Therefore, beyond these general concepts of Hygiene and Westernization, it is critical to understand and clarify exactly which germs the recent modernization has removed from our ‘microbiome’ (i.e., microbes we are in contact with). Consistent with the HH, it would be the disappearance of these organisms from our environment, depriving us of their immunomodulatory properties, which would have contributed to the recent outbreak of immune-mediated disorders in Western countries.

The evolution of our microbial environment through the millennia was elegantly analyzed by Rook [24,60], who refers to these organisms as ‘Old Friends’. According to him, the relevant microorganisms with a probable immunoregulatory role are those that were part of our natural environment and with which we co-evolved and lived in close contact since periods as far back as the Paleolithic, until a few decades ago, when our society was still largely rural, living on farms and in contact with animals [61]. So far, we accepted and tolerated these organisms in our body, cohabiting in relative harmony with them, the latter being ultimately largely removed from our modern urbanized environment. In view of this, we are primarily concerned with helminthes and microbes acquired by oro-fecal transmission or that can induce an asymptomatic carrier state (hepatitis A virus, *Mycobacteria*, *Toxoplasma*, *Helicobacter pylori*) as well as those composing the commensal (cutaneous, intestinal, oro-pharyngeal, genitourinary) and the environmental flora (present in mud, water, soil, plants, animals). Common infections of childhood were often considered as part of the relevant microbes in the HH. However, most childhood viruses, such as measles, mumps, and chickenpox, are most frequently not protective against chronic inflammatory disorders [28,62,63], and even often trigger them [64]. Unlike other previously mentioned microorganisms, these have not peacefully co-evolved with us, and either have a harmful effect that can kill the host, or induce a strong immune response. Called by Rook ‘crowd infections’ [60], these viruses require large populations and close contacts to persist, but do not exert a beneficial role in our organism and therefore, unlike other microbes, did not coexist with us.

### Evolution of our microbial environment in the Western world

It is of interest to attempt to understand what developments in our lifestyle have led to the dramatic changes in our contact with these ancestral organisms within a few decades.

First, urbanization and migration of populations to cities, as well as public health measures, such as control of food production, water quality, and advances in sanitation and health care, have significantly reduced or almost eradicated some infections in Western countries, particularly helminthic infections [65,66], malaria, mycobacterial infections [67], and hepatitis A. As indicated above, epidemiological data have inversely correlated the eradication of some of these infections, especially helminthic infections and malaria, with an increase in the prevalence of immune-mediated diseases in Western countries.

Otherwise, the composition of our gut microbiota strongly depends on our environment, mainly on our microbial contacts as well as many other factors that can modulate it. It has been demonstrated that the gut microbiota plays a critical role in regulating the immune response [68,69]. Thus, any factor causing a dysregulation of the microbiota can affect the balance of our immune system and thereby promote the development of chronic inflammatory diseases [70-72]. Hence, population migration from rural areas in contact with animals and environmental flora to more sanitized urban areas has affected the microbiota diversity [73,74], thereby likely favoring immune-mediated disorders. Interestingly, several studies [17,75,76] have suggested that the protective effect of large families or owning animals on atopic diseases reported in epidemiological studies could be partially related to an increase in gut microbiota diversity and richness. This may explain the pioneering observations reported by Strachan [16] on the protective effect of a large number of siblings on the occurrence of allergic disorders. Similarly, a Western diet [77-80], widespread use of antibiotics [81], and birth by caesarean section [82] are well-established factors disrupting intestinal microbiota. Several studies have demonstrated that exposure to antibiotics at an early age may cause dysbiosis, increasing the risk of subsequent allergic disorders [36,83,84]. In addition, caesarean birth has been associated with a higher risk of asthma [85], T1D [86], MS [87], and celiac disease [88,89].

Thus, eradication of most of these ‘Old Friends’ from our environment may have contributed to the recent outbreak in inflammatory disorders in Western countries. To better explain this inverse correlation, it is important to unravel the wide immunomodulatory effects of these microorganisms on their host’s immune system. To date, helminthes have provided the greatest information to specify the protective mechanisms developed in the host, most data being derived from animal models.

### An example of immunomodulation by infectious agents: about helminthes

Helminthes are eukaryotic parasitic worms. In 2008, it was estimated that about 37% of the world’s population was infected with helminthes, mainly in developing countries, helminthiasis now being rare in industrialized countries [66]. Helminthes’ classification is based on numerous factors, including the external and internal morphology of egg, larval, and adult stages. These parasites are divided into two phyla: Platyhelminthes (flatworms), including both trematodes (flukes) and cestodes (tapeworms), and Nemathelminthes, including only one class, namely nematodes (roundworms) [90]. Helminthes most frequently live in the gastrointestinal tract of their host, but may also colonize other organs. It is worth noting that helminthes have co-evolved with their host for millennia; their goal is not to kill their host but to survive as long as possible by creating a state of tolerance. To achieve this, helminthes are able, through various mechanisms, to finely modulate the host immune system to prevent an activation that may lead to their elimination, while not causing too deep an immunosuppression which would cause the host to die from infection. This immunomodulation, by avoiding an excessive activation of the immune system, contributes to host protection against inflammatory disorders.

**Figure 1:**
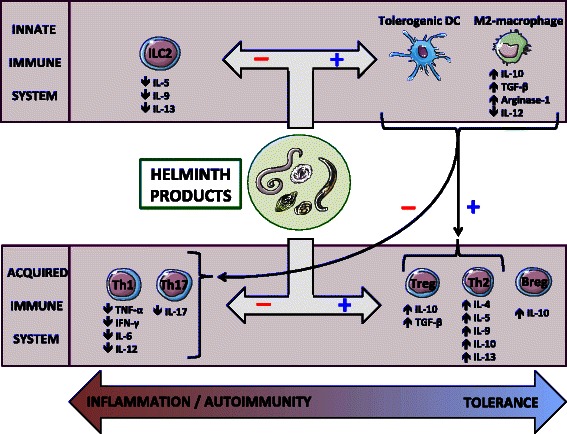
**Immunoregulatory effects of helminthes on the immune system.** Helminthes exert their immunoregulatory actions by modulating cells of both the innate and adaptive immune system. Regarding T-cells, helminthes may promote a Th2-type response and down-regulate Th1/Th17 differentiation, leading to increased Th2-type cytokine (IL-4, IL-5, IL-9, IL-10, IL-13) and decreased Th1/Th17-type cytokine (TNF-α, IFN-γ, IL-6, IL-12, IL-17) secretion. Furthermore, worms’ products enhance Treg cell proliferation, the latter hampering Th1/Th2/Th17 polarization mainly through the secretion of IL-10 and TGF-β. Helminthes also promote a regulatory phenotype of B-cells, DCs, and macrophages. Both tolerogenic DCs and regulatory M2-macrophages contribute to switching from a Th1/Th17 to a Th2/Treg profile. Finally, these parasites may hamper the proliferation of ILC2, a subset of innate immune cells responsible for allergic responses. Thus, helminthes create a tolerant environment ensuring their own survival but also protecting the host from immune-mediated conditions by limiting excessive inflammatory and autoimmune phenomena. We declare that this figure is original. Breg, B-regulatory cell; DC, Dendritic cell; IFN, Interferon; IL, Interleukin; ILC2, Type-2 Innate lymphoid cell; TGF, Transforming growth factor; Th, T-helper cell; TNF, Tumor necrosis factor; Treg, T-regulatory cell.

Numerous studies in animal models have highlighted the intricate mechanisms by which helminthes hamper the host’s immune response. This includes promotion of T-helper-2 (Th2) and inhibition of Th1/Th17 differentiation, amplification of T-regulatory (Treg) and B-regulatory (Breg) cells and type 2-macrophages, orientation of dendritic cells (DCs) towards a tolerogenic phenotype, downregulation of type-2 innate lymphoid cells (ILC2), and modulation of the gut microbiota [24,91-95]. Helminthes’ immunoregulatory effects are illustrated in Figure 1.

#### Th1 and Th2 cells

Naïve T-cells may differentiate into either Th or Treg cells. Regarding Th cells, the old paradigm opposed two main responses, Th1- and Th2-type responses, down-regulating each other [96]. Thus, the Th1 response is demonstrated to be particularly involved in the pathogenesis of autoimmune diseases and is associated with expansion of Th1 cells as well as secretion of pro-inflammatory cytokines, including interleukin (IL)-6, IL-12, interferon (IFN)-γ, and tumor necrosis factor (TNF)-α. Conversely, the Th2 response plays a central role in atopic disorders and is characterized by expansion of mast cells, eosinophils, and increased levels of IL-4, IL-5, IL-9, IL-10, IL-13, and IgE [97]. The original vision derived from experiments in mouse models of autoimmune diseases infected with helminthes [98] suggested that helminthes were acting on the Th1/Th2 balance by promoting Th2 and inhibiting Th1 polarization, resulting in a protective effect against Th1-mediated autoimmune diseases. However, this conception did not explain the epidemiology of the HH showing a protective effect of helminthes also with regard to allergic diseases. Indeed, by promoting a Th2 differentiation, helminthes should increase atopic disorders. Therefore, it has become increasingly obvious that other cellular actors are involved in immunoregulation mediated by helminthes such as Th17 and Treg cells. Th17 cells are a recently-defined subset of Th cells primarily secreting IL-17 and whose role in autoimmune diseases has been widely documented [99,100]. Treg cells [101], for their part, have been found to control both Th2-mediated allergy [102] and Th1/Th17-mediated inflammatory disorders, mainly through the secretion of IL-10 and TGF-β [103].

Many experiments have demonstrated that helminthes are able to modulate the host’s immune response by inhibiting Th17 differentiation [104-108], promoting Th2 relative to Th1 polarization [104,106,107,109-114], and chiefly by enhancing Treg cell proliferation and secretion of IL-10 and TGF-β [104-107,110-113,115], resulting in an overall control of Th1/Th2/Th17 responses [92]. The modulation of the T-cell profile contributes to the protective effect of parasites against both allergic and autoimmune diseases. For example, prior infection of Dark Agouti rats – prone to developing experimental autoimmune encephalitis (EAE), the most commonly used animal model of MS – with the nematode *Trichinella spiralis* (*T. spiralis*) [104], reduced clinical and histological manifestations of the disease. It was accompanied by a down-regulation of Th1 (IFN-γ) and Th17 cytokines (IL-17) and an up-regulation of Th2/Treg cytokines (IL-4, IL-10) as well as Treg cell proliferation. Moreover, transfer of splenic T-cells from *T. spiralis*-infected rats into non-infected EAE rats led to an improvement of EAE and, in some cases, protection from disease development. Similarly, the excretory-secretory (ES) product of the rodent filarial nematode *Acanthocheilonema vitae* (*A. vitae*), namely ES-62, was found to suppress collagen-induced arthritis (CIA) severity and progression in mice by inhibiting Th1- and Th17-associated cytokines (TNF-α, IFN-γ, IL-6, IL-17) [108,114]. *Schistosoma mansoni* (*S. mansoni*) infection also significantly reduced the severity of mice CIA by lowering pro-inflammatory cytokines (IFN-γ, TNF-α, IL-17A) and rising Th2/Treg cytokines (IL-4, IL-10) [107].

#### Dendritic cells

DCs are recognized as a pivotal link between the innate and adaptive immune system. Their main function is to capture, process, and present antigens to T-cells. Pattern recognition receptors, including Toll-like receptor (TLR) and C-type lectin receptor families, are particularly important to this process [116]. The activation status of DCs is crucial in T-cell polarization [117]. Thus, DCs may adopt a ‘tolerogenic’ phenotype opposing the ‘immunogenic’ phenotype, able to initiate Th2 and Treg responses [118]. These tolerogenic DCs have been shown to be essential in the prevention of autoimmunity [116]. Helminthes’ products are able to modulate DC signaling to direct their differentiation toward a tolerogenic phenotype [116,119]. This modulation is partly mediated by the binding and recognition of parasitic products by TLR and C-type lectin receptors [120]. In the Dark Agouti rat model of EAE reported above, the authors reproduced the beneficial results obtained with *T. spiralis* infection [104] by injecting non-infected rats with DCs stimulated with ES products released from encysted muscle larvae of *T. spiralis* (ES-L1) 7 days before EAE induction [121]. ES-L1-stimulated DCs increased the CD4+ CD25+ Foxp3+ Treg cell population as well as IL-4, IL-10, and TGF-β production, and decreased IFN-γ and IL-17 levels. Further, two studies [122,123] used a Rag IBD mice model (Rag mice lack functional T- and B-cells) where animals are reconstituted with IL-10−/− T-cells; IL-10 being a key immunoregulatory cytokine, IL-10−/−−deficient mice develop spontaneous chronic colitis. In these studies, *Heligmosomoides polygyrus* (*H. polygyrus*), an intestinal helminth, was demonstrated to prevent and reverse intestinal inflammation, either by direct infection of IBD mice [122] or by transfer of DCs from infected-mice to IBD mice [123]. Thus, *H. polygyrus* was shown to mediate IBD protection by altering DC function in a regulatory phenotype. *H. polygyrus*-exposed tolerogenic DCs rendered T-cells hyporesponsive and inhibited IFN-γ/IL-17 responses.

#### B-regulatory cells

Helminthes may promote the proliferation of B-regulatory (Breg) cells [124,125]. Besides conventional B-lymphocytes responsible for T-cell activation and antibody production, a specific subset of B-cells has recently been highlighted. Breg cells have been shown to negatively regulate the immune response by producing regulatory cytokines, mainly IL-10, and through direct interaction with pathogenic T-cells. The regulatory function of Breg cells has since been demonstrated in various pathological conditions including autoimmune diseases [126,127]. In CIA mice, the ES product of *A. vitae*, ES-62, in addition to modulating T-cell response as described above, is able to restore IL-10-producing Breg cell levels while decreasing plasma cell infiltration in the joints [128]. Similarly, the generation of Breg cells by helminthes was reported in MS [129] and IBD [130] mice models. Interestingly, Correale et al. [131] demonstrated that helminth-infected MS patients created a Breg cell population producing high amounts of IL-10 as well as neurotrophic factors involved in the growth and development of neurons. These patients exhibited significantly lower clinical and radiological disease activity when compared to non-infected patients.

#### Innate immune cells

Besides modulating T-cells, DCs, and Breg cells, helminthes were also shown to exert their immunomodulatory effects by manipulating innate immune cells, especially alternatively activated or M2-macrophages and ILC2.

In response to diverse stimuli, macrophages may undergo classical M1 activation (stimulated by TLR ligands or IFN-γ) or alternative M2 polarization (stimulated by IL-4/IL-13 axis). M2-macrophages, unlike M1-macrophages, have a low expression of IL-12, high expression of IL-10, TGF-β, and arginase-1, and exhibit anti-inflammatory and immunosuppressive functions [132]. Thus, by using a mice model with IL-4R−/− macrophages it was shown that generation of M2-macrophages is essential for mice survival during schistosomiasis through their inhibitory effects on Th1 response [133]. Further, *Litomosoides sigmodontis* (*L. sigmodontis*) infection in mice recruited a F4/80+ population of alternatively activated macrophages that potently inhibited Ag-specific CD4+ T-cell proliferation [134]. Moreover, the type-1 cystatin derived from the liver fluke *Clonorchis sinensis* (*C. sinensis*) significantly reduced intestinal inflammation by recruiting IL-10-secreting macrophages in a dextran-sodium-sulfate (DSS)-induced colitis mice model [135].

ILCs are a recently described population of lymphocytes that lack B- and T-cell lineage markers and are divided into three subtypes: ILC1, which secrete IFN-γ; ILC2, which secrete IL-5 and IL-13; and ILC3, which secrete IL-17 and IL-22. ILCs play a role in host anti-helminth protective immunity and are able to initiate allergy. The cytokines IL-25, IL-33, and thymic stromal lymphopoietin (TSLP) have been shown to drive ILC2 expansion [136,137]. The immunomodulatory effects of helminthes on this cell subtype remain unclear. However, McSorley et al. [138] recently highlighted the potential immunomodulatory effect of helminthes on ILC in an airway allergy mice model. *H. polygyrus* ES products were found to suppress ILC2 response by blocking IL-33 secretion, thereby abolishing the allergic response to allergens. Further studies are needed to clarify the role of these innate immune cells in the protective effect induced by helminthes.

#### Microbiota regulation

Apart from its actions on the immune system, helminthes may modulate the bacterial composition of intestinal flora, promoting the growth of gut microorganisms typically considered to be ‘probiotics’ [139,140]. As mentioned above, the microbiome plays an essential role in regulating the immune system [68,69] and disrupting the balance of the gut microbiome may promote the development of autoimmune diseases [70-72]. Additionally, as previously discussed, many factors, to date, have been involved in the modulation of the intestinal microbiota and may in this way prevent the development of inflammatory disorders. Similarly, by maintaining the microbiome balance, helminthes likely contribute to the prevention of immune-mediated disorders.

The mechanisms through which some microorganisms can manipulate the host’s immune response to ensure their own survival are complex and interlinked, and will need to be specified in further studies. However, it is likely that immunomodulatory strategies depend on both the pathogen involved and host factors such as genetic background and the local microenvironment. Meanwhile, the exceptional immunomodulatory properties of these microorganisms, particularly helminthes, which are the best characterized to date, led to their successful application in autoimmune disease treatment in both animal models and clinical trials, thereby providing evidence of the HH and paving the way to a new therapeutic potential.

### Proof of concept of the HH: helminth therapy

The recognition of the extensive immunoregulatory properties of numerous microorganisms from our environment, especially helminthes, initially suggested in the HH and since then widely demonstrated, led, in the 1990s, to their therapeutic application in experimental models and clinical studies of several immune-mediated conditions, including IBD, MS, RA, T1D, celiac disease, Grave’s disease, and psoriasis. Various species of helminthes and different approaches were evaluated, including colonization by helminthes larvae, oral administration of helminthes ova, and the use of helminth-derived antigens. Helminth-derived therapies, as detailed below, were found to both prevent or delay the onset and reduce the severity of autoimmune diseases in both animal models and clinical trials. These experiments are summarized in Table 1.

**Table 1:** **Experimental and clinical studies of helminth-derived therapies in autoimmune diseases**

**Inflammatory bowel disease**	Mice	*Trichinella spiralis* [141,147,153]	Attenuates DNBS-induced colitis
			↑ IL-4, IL-13, TGF-β and ↓ IFN-γ, IL-1β, MPO activity, iNOS expression
		*Schistosoma mansoni* [142,143,145]	Prevents or attenuates TNBS- and DSS-induced colitis
			↑ IL-4, IL-10, F4/80+ macrophages and ↓ IFN-γ
		*Heligmosomoides polygyrus* [122,123]	Prevents Rag IL-10−/− T-cell transfer model of colitis
			↑ tolerogenic DC and ↓ IFN-γ, IL-17
		*Hymenoleptis diminuta* [144,148]	Attenuates DNBS-induced colitis
			↑ IL-10
		*Schistosoma japonicum* [146]	Attenuates TNBS-induced colitis
			↑ IL-4, IL-5, IL-13, Treg, and ↓ IFN-γ
		*Clonorchis sinensis* [135]	Attenuates DSS-induced colitis
			↑ IL-10, IL-10+ F4/80+ macrophages and ↓ TNF-α
	Human	*TSO* [149-152]	CD: open-label study: 79.3% responded, 72.4% remitted at 24 weeks
			UC: placebo-controlled trial: 43.3% responded 12 weeks versus 16.7% in placebo group; no significant difference in remission rates
			CD: phase 2 TRUST-I trial: no significant differences between TSO and placebo groups
		*Necator americanus* [155]	Improvement CDAI in 5/9 patients at 20 weeks and 3/5 at 45 weeks
			Mild adverse events
**Multiple sclerosis**	Mice	*Schistosoma mansoni* [158,160,162]	Reduces incidence and attenuates EAE
			↑ IL-4, IL-10, TGF-β and ↓ IFN-γ, TNF-α, IL-12, and CNS inflammatory cell infiltration
		*Trichinella spiralis* [104,121,157,163]	Attenuates EAE
			↑ IL-4, IL-10, TGF-β, Treg, tolerogenic DC and ↓ IFN-γ, IL-17
		*H. polygyrus* [129]	Prevents EAE by transfer of B-cells from IL-10−/− infected mice
		*Trichinella pseudospiralis* [106]	Delays and attenuates EAE ↑ IL-4, IL-5, IL-10 and ↓ TNF-α, IFN-γ, IL-1β, IL-6, IL-17
		*Schistosoma japonicum* [164]	Attenuates EAE
			↑ IL-4 and ↓ IFN-γ and CNS inflammation
		*Fasciola hepatica* [161]	Attenuates EAE ↑ IL-10, tolerogenic DC, M2-macrophages, IL-10 secreting T-cells and ↓ IFN-γ, IL-17
		*Taenia Crassiceps* [159]	Attenuates EAE
			↑ IL-4, IL-10, M2-macrophages and ↓ TNF-α, IL-17, iNOS expression and CNS inflammation
	Human	*Hymenoleptis nanan*, *Trichuris trichura*, *Ascaris lumbricoides*, *Strongyloides stercolaris*, *Enterobius vermicularis* [131,165,166]	12 naturally infected MS patients and 12 controls, follow-up 7.5 years
			↓ relapses, disability scores, MRI activity, and ↑ IL-10, TGF-β, Treg, Breg and ↓ IFN-γ, IL-12 in infected patients
			Anti-helminthic treatment ↑ clinical and radiological activity
		*TSO* [167]	5 patients with relapsing-remitting MS
			↓ mean number of new MRI lesions
**Rheumatoid arthritis**	Mice	*Acanthocheilonema vitae* (ES-62) [108,128,169,171-173]	Reduces incidence and attenuates CIA
			↑ IL-10, late IL-22, tolerogenic DC, Breg and ↓ TNF-α, IFN-γ, IL-6, IL-17, early IL-22, IgG2a
			Immunomodulatory effects related to PC moiety
		*Schistosoma mansoni* [107]	Attenuates CIA
			↑ IL-4, IL-10 and ↓ TNF-α, IFN-γ, IL-1β, IL-6, IL-17A, IgG2a
		*Hymenoleptis diminuta* [174]	Attenuates Freund’s complete adjuvant-induced arthritis
			Protection abrogated in mice lacking T- and B-cells or IL-4Rα or IL-10
		*Fasciola.hepatica* [175]	Reduces incidence and attenuates CIA
			↑ IL-10, TGF-β, tolerogenic DC, Treg and ↓ TNF-α, IL-17A, IgG2a
		*Schistosoma japonicum* [176]	Attenuates CIA
			↑ IL-10, Treg, IgG1 and ↓ TNF-α, IFN-γ, IL-1β, IL-6 Th-17 cells, IgG2a
		*Heligmosomoides polygyrus* and *N. brasiliensis* [177]	Reduces incidence and attenuates spontaneous arthritis in MRL/lpr mice
			↑ IL-4, IgG1
**Type-1 diabetes**	Mice	*Schistosoma mansoni* [111-113,178,179]	Reduces incidence or prevents diabetes in NOD mice
			↑ IL-4, IL-5, IL-10, IL-13, TGF-β, tolerogenic DC, Treg, V alpha 14i NKT cells
			Prevents class switch from IgM to IgG anti-insulin autoantibodies
			ω1 glycoprotein secreted by *S. mansoni* ova responsible for its effects
		*Heligmosomoides polygyrus* [109,180]	Prevents and reduces severity of diabetes in NOD mice
			↑ IL-4, IL-10, IL-13, Treg and ↓ pancreatic insulitis
		*Trichinella spiralis* [109]	Prevents diabetes in NOD mice
			↑ IL-4 and ↓ pancreatic insulitis. No change in IL-10 and IFN-γ
		*Litomosoides sigmodontis* [110,115]	Prevents diabetes in immunocompetent and IL-4 deficient NOD mice
			↑ IL-4, IL-5, IgG1, Treg and ↓ pancreatic insulitis
		*Dirofilaria immitis* [181]	Prevents diabetes in NOD mice
			↑ IgE. Prevents class switch from IgM to IgG anti-insulin autoantibodies
**Celiac disease**	Human	*Necator americanus* [183,184]	No significant change in symptom severity at the gluten challenge following treatment
			↓ IFN-γ, IL17A
**Systemic lupus erythematosus**	Mice	*Schistosoma mansoni* [185]	Change glomerulonephritis phenotype from diffuse proliferative to membranous pattern
			↑ IL-4, IL-5, IL-10, and TGF-β
**Graves’ disease**	Mice	*Schistosoma mansoni* [186]	Prevent Grave’s disease development
			↓ IFN-γ, IgG2a, anti-TSHR antibodies
**Psoriasis**	Mice	*Schistosoma mansoni* [187]	Prevent psoriatic skin lesions in fsn/fsn mice
			↑ IL-13 and ↓ IFN-γ

Breg, B-regulatory cells; CD, Crohn’s disease; CDAI, Crohn’s disease activity index; CIA, Collagen-induced arthritis; CNS, Central nervous system; DC, Dendritic cell; DNBS, Dinitrobenzene sulfonic acid; DSS, Dextran-sodium-sulfate; EAE, Experimental autoimmune encephalitis; iNOS, Inducible nitric oxide synthase; IFN, Interferon; IL, Interleukin; MPO, Myeloperoxidase; MRI, Magnetic resonance imaging; MS, Multiple sclerosis; NKT cells, Natural killer T-cells; NOD, Non-obese diabetic; PC, Phosphorylcholine; TGF, Transforming growth factor; Th, T-helper cells; TNBS, Trinitrobenzene sulfonic acid; TNF, Tumor necrosis factor; Treg, T-regulatory cells; TSHR, Thyroid stimulating hormone receptor; TSO, *Trichuris suis* ova; UC, Ulcerative colitis.

### Inflammatory bowel disease (IBD)

Strong experimental data using helminthes in various murine models of colitis have suggested the benefit of helminth therapy in IBD [122,123,135,141-148]. Thus *S. mansoni* soluble egg antigen (SEA) [142] or cercariae [143,145] exposure were shown to significantly attenuate trinitrobenzene sulfonic acid (TNBS)-induced [142,143] or DSS-induced [145] colitis in mice by enhancing IL-4 and IL-10 expression, decreasing INF-γ levels [142,143], or through induction of F4/80+ macrophages [145]. Infection of mice with either *T. spiralis* cercariae [141] or antigens [147] prior to dinitrobenzene sulfonic acid (DNBS)-colitis induction reduced its severity and was correlated with higher IL-4, IL-13, and TGF-β production and down-regulation of IFN-γ, IL-1β, myeloperoxidase activity, and inducible nitric oxide synthase expression. Similarly, *H polygyrus* [122,123], *Hymenoleptis diminuta* (*H. diminuta*) [144], *Schistosoma japonicum* (*S. japonicum*) [146], and *C. sinensis* [135] were found to attenuate or prevent the Rag IL-10−/− T-cell transfer model of colitis and DNBS-, TNBS-, and DSS-induced colitis, respectively, through mechanisms including promotion of Treg cells, Th2-cytokines (IL-4, IL-5, IL-10), tolerogenic DCs, and M2-macrophages, as well as inhibition of IFN-γ and IL-17 secretion.

These encouraging results have led to several clinical trials [149-152] evaluating the safety and therapeutic potential of helminth therapy, mainly *Trichuris suis* (*T. suis*) ova (TSO) in IBD patients. *T. suis* is a pig whipworm able to colonize a human host only for a short period of time. Following an initial study [149] suggesting that TSO given orally could be a safe and effective treatment of IBD, Summers et al. conducted two trials; an open-label study in 29 patients suffering from active CD as defined by a Crohn’s disease activity index (CDAI) ≥220 [150], and a randomized double blind placebo-controlled trial in 54 patients with active UC, defined by an ulcerative disease activity index (UCDAI) ≥4 [151]. All CD patients ingested 2,500 TSO every 3 weeks for 24 weeks; UC received either 2,500 TSO or placebo orally at 2-week intervals for 12 weeks. At the studies’ conclusion, 79.3% of CD patients responded (decrease in CDAI >100 points or CDAI <150) and 72.4% remitted (CDAI <150), whereas, although UC remission rates between the two groups were not significantly different, improvement of the UCDAI occurred in 43.3% of patients with ova treatment compared with 16.7% given placebo. No side effects were reported in either study. Although *T. suis* immunoregulatory mechanisms were not studied in these trials, it is assumed that this involves the modulation of Th1, Th2, Treg, and Th17 subsets as suggested in murine models. In October 2013, the TRUST-I phase 2 clinical trial [153] evaluating TSO treatment (7,500 ova every 2 weeks for 12 weeks) versus placebo in 250 moderate-to-severe CD patients failed to reach its primary (100-point CDAI decrease) and key secondary (achieving CDAI <150) endpoints. Despite these discouraging results, the authors assumed, according to subgroup analyses, that the effectiveness of TSO could be higher in severe patients (CDAI >290). A number of clinical trials using TSO in IBD patients have been performed (listed in [95,154]), and the human hookworm *Necator americanus* (*N. americanus*) has been suggested as an alternative to *T. suis* since it is easier to use due to longer-lasting effects [155] and appears to be well tolerated [156].

### Multiple sclerosis (MS)

Many studies [104,106,121,129,157-163] conducted in EAE mice, the main animal model of MS, have highlighted the interest of helminth-derived therapies in this disease. Indeed, prior treatment of mice before EAE induction with either *S. mansoni* ova [160], cercariae [158], or antigen [162] significantly reduced the incidence as well as the severity of EAE as measured by clinical scores and central nervous system (CNS) inflammation. This protective effect was associated with decreased IL-12, IFN-γ, and TNF-α secretion and higher IL-4, IL-10, and TGF-β levels in periphery. It is of interest to note that increased IL-4-secreting neuroantigen-specific T-cells and reduced macrophage and CD4+ T-cell infiltration were observed in the CNS of infected mice as compared to controls. Similarly, pretreatment of EAE Dark Agouti rats with *T. spiralis* ES products (ES-L1) [104,163] ameliorated the clinical and histological severity of induced EAE in a dose-dependent manner. The mechanisms involved an inhibition of Th1 and Th17 cytokines (IFN-γ, IL-17) and a promotion of Th2 cytokines (IL-4, IL-10) and TGF-β, as well as induction of Treg cells. Transfer of splenic T-cells from *T. spiralis*-infected rats into EAE rats led to protection from disease development in some cases. As indicated above, injection of DCs stimulated with *T. spiralis* ES-L1 products 7 days before EAE induction [121] was also found to ameliorate EAE by increasing IL-4, IL-10, TGF-β, and Treg levels and decreasing IFN-γ and IL-17 secretion, both at the systemic level and in target organs. Further, several studies have demonstrated the benefit of *H. polygyrus* [129], *Trichinella pseudospiralis* [106], *S. japonicum* [164], *Fasciola hepatica* [161], and *Taenia crassiceps* [159] infections in preventing or delaying the onset of EAE and improving its severity. These effects result from inhibition of Th1- and Th17-responses with lower IFN-γ, TNF-α, IL-6, and IL-17 secretion, reduction of CNS inflammatory infiltrates, enhanced Th2 cytokine production (IL-4, IL-10), and proliferation of Breg, Treg, M2-macrophage, and tolerogenic DC populations.

To date, few studies have evaluated the therapeutic potential of helminthes in MS patients. The most noteworthy observations were reported by Correale et al. [131,165,166] through several prospective studies comparing a series of 12 patients with MS naturally infected with different species of helminthes (*Hymenoleptis nanan*, *Trichuris trichura*, *Ascaris lumbricoides*, *Strongyloides stercolaris*, and *Enterobius vermicularis*) with 12 non-infected MS patients as well as with infected patients without MS and healthy subjects. The authors assessed the clinical, radiological, and immunological characteristics of each group of patients with a 4.6-year follow-up extended to 7.5 years in a later report. During the initial 4.6-year follow-up period [165], parasite-infected MS patients showed a significantly lower number of relapses, reduced disability scores, and lower magnetic resonance imaging activity compared to uninfected MS subjects. Infected patients showed higher IL-10- and TGF-β- and lower IL-12- and IFN-γ-secreting cell levels. Moreover, Treg and IL-10-secreting Breg cells were significantly increased in parasite-infected patients compared to other groups [131]. Interestingly, these Breg cells were also found to produce greater amounts of brain-derived neurotrophic factor and nerve growth factor, raising the possibility that these cells may exert a neuroprotective effect on the CNS [131]. Anti-helminthic treatment of 4 of the 12 patients treated due to gastrointestinal symptoms [166] led to a significant rise in clinical and radiological MS activities and in the number of IFN-γ- and IL-12-secreting cells together with a fall in the levels of Treg cells and TGF-β- and IL-10-secreting cells, which became evident 3 months after anti-helminthic treatment began.

A safety phase 1 study (phase 1 Helminthes-induced Immunomodulatory Therapy – HINT-1) [167] inoculating 2,500 TSO orally every 2 weeks for 3 months in five relapsing-remitting MS patients reported mild gastrointestinal side effects. The mean number of new MRIlesions fell from 6.6 at baseline to 2.0 after 3 months of TSO administration and rose again to 5.8 at 2 months after the end of the study. In view of these encouraging results, several clinical trials are already underway or planned, including an extension of HINT-1 as well as numerous studies evaluating TSO or dermally-administrated hookworm *N. americanus* as therapies in MS patients (listed in [95,154,168]).

### Rheumatoid arthritis (RA)

Several helminthic products with immunomodulatory properties have been studied in mice models of RA, the filarial-derived glycoprotein ES-62 being the best characterized [169]. However, so far, no helminth-derived molecules have been used in RA clinical trials.

ES-62 is a tetrameric phosphorylcholine (PC)-containing glycoprotein secreted by the rodent filarial nematode *A. vitae* first described in 1989 [170]. A decade ago, this glycoprotein was demonstrated to significantly reduce the initiation, severity, and progression of CIA in DBA/1 mice, a murine model of RA [114]. Since then, its protective mechanisms in CIA mice have been elucidated by Harnett et al. through several experiments [108,128,171-173]. ES-62 inhibits Th1- and Th17-responses resulting in decreased levels of IFN-γ, TNF-α, IL-6, and IL-17 both in draining lymph nodes and joints of CIA animals. Regarding Th-17 response, ES-62 down-regulates IL-17 secretion by both innate (γδ T cells) and adaptive (Th17 CD4+ cells) cells via DC-dependent and independent mechanisms [108]. It decreases collagen-specific IgG2a antibody production and reduces effector B-cells, particularly plasma cell activation, proliferation, and joint infiltration. Conversely, ES-62 was found to up-regulate IL-10 secretion by splenocytes and to restore IL-10-secreting Breg cell levels in CIA mice [128]. IL-22 plays a dual role in CIA, being pathogenic during the initiation phase while acting to resolve inflammation and joint damage during established disease. Exposure to ES-62 *in vivo* suppressed the early peak of IL-22 but induced strong expression at later time points, serum levels of IL-22 correlating inversely with articular scores [172]. Finally, ES-62 is also able to modulate DCs to promote a Th2 response. Interestingly, most of the anti-inflammatory actions of ES-62 in CIA have been shown to be related to its PC moiety [171]. Indeed, the whole ES-62 molecule and a PC-ovalbumin conjugate successfully reduced disease severity by suppressing Th1 cytokine production while a PC-free recombinant form of ES-62 failed to prevent CIA progression [173]. Furthermore, a sulfone-containing PC analogue (11a) was designed and demonstrated to be effective in protecting DBA/1 mice from developing CIA [173]. Apart from *A. vitae* secretory product, several helminthes, including *S. mansoni* [107], *H. diminuta* [174], *F. hepatica* [175], *S. japonicum* [176], *H. polygyrus*, and *Nippostrongylus brasiliensis* [177], have been demonstrated to effectively prevent RA-like disease in mice models through inhibition of Th1/Th17 cytokine secretion, induction of tolerogenic DCs, and promotion of Treg cell proliferation.

### Type-1 diabetes (T1D)

No clinical trial using helminth-derived therapies has so far been conducted in T1D patients. However, a large body of experimental data from non-obese diabetic (NOD) mice [109-113,115,178-181], the murine model of T1D, suggest their effectiveness, especially for *S. mansoni* [111-113,178,179].

Cooke et al. [178] were the first to suggest *S. mansoni* infection as a preventive treatment in NOD mice. Infection with *S. mansoni* cercariae or SEA significantly reduced the spontaneous incidence of diabetes and prevented the class switch from IgM to IgG anti-insulin autoantibodies normally seen in most NOD mice as they approach overt diabetes. The authors subsequently reproduced this protective effect in a series of experiments by specifying its mechanisms [111-113,179]. *S. mansoni* SEA completely prevented the occurrence of T1D when injected to 4-week-old NOD mice [111] by expanding Treg cells in a TGF-β-dependent manner and Th2 cells with increased secretion of IL-4, IL-5, IL-10, and IL-13. Moreover, T-cells from SEA-treated mice exhibited a reduced ability to transfer diabetes to NOD-severe combined immunodeficiency recipients. NOD mice are known to have deficiency in V alpha 14i NKT cells, the expansion of this population preventing diabetes onset [182]. *S. mansoni* SEA increased the number of V alpha 14i NKT cells and induced functional changes in DCs, found to secrete more IL-10 and less IL-12. Recently, ω-1, one of the two major glycoproteins secreted by *S. mansoni* ova, was demonstrated to be responsible for its effects [113]. Several studies also evidenced the efficacy of *H. polygyrus* [109,180], *T. spiralis* [109], *L. sigmodontis* [110,115], and *Dirofilaria immitis* (*D. immitis*) recombinant antigen [181] to completely prevent the occurrence of diabetes in NOD mice especially by eliciting a Th2-type response and promoting the proliferation of Treg cells, thereby markedly inhibiting pancreatic insulitis.

### Other immune-mediated diseases

Experimental and clinical data evaluating helminth therapy in other inflammatory conditions are scarce.

Regarding celiac disease, a double-blinded placebo-controlled study [183] explored the effects of *N. americanus* cutaneous inoculations in 10 patients compared with 10 non-infected patients. Inoculations of 15 third-stage larvae were performed at weeks 0 and 12, and a 5-day oral gluten challenge was undertaken at week 20. No significant reduction in symptom severity was seen in infected subjects compared to non-infected patients. However, immunological data from the clinical trial analyzed in a subsequent study [184] found that basal Th1- and Th17-responses were inhibited in the duodenum of hookworm-infected patients with decreased IFN-γ and IL-17A secretion. The authors hypothesized that the infective dose of hookworms used in the trial may be insufficient to effectively suppress the immunopathology of celiac disease.

In SLE, a recent study [185] analyzed, for the first time, the effects of infection of MRL/lpr lupus mice with the trematode *S. mansoni.* The infection completely changed the phenotype of glomerulonephritis in MRL/lpr mice, switching from a severe diffuse proliferative Th1-mediated pattern towards a membranous Th2-mediated nephritis associated with a better prognosis. This effect was associated with a modulation of the cytokine profile shifting from a Th1 to a Th2 polarization with increased rates of IL-4, IL-5, IL-10, and TGF-β.

In Grave’s disease, prophylactic use of *S. mansoni* product homogenates in a mouse model prior to disease induction prevented its development through Th2 polarization [186]. This was associated with decreased IgG2a subclass anti-thyroid stimulating hormone receptor antibody production, lower IFN-γ levels, and enhanced Treg proliferation.

Finally, one experiment [187] conducted on the fsn/fsn mouse model of psoriasis demonstrated the efficacy of subcutaneous *S. mansoni* LNFPIII glycan treatment to prevent the appearance of psoriatic skin lesions as compared with control mice. Skin cells from LNFPIII-treated mice secreted lower amounts of IFN-γ and increased levels of IL-13, evidencing a shift toward a Th2-type response. It is noted that several psoriasis clinical trials using TSO are planned [95,154].

### Future perspectives and challenges

The emerging potential of helminth-derived therapy in autoimmune diseases is raising growing interest. The encouraging data from mouse models and early clinical studies have led to an increase in the number of phase 1 and 2 clinical trial projects in IBD, MS, RA, psoriasis, and celiac disease [154]. Recently, we have successfully employed helminthes’ PC derivatives to treat colitis using a prophylactic protocol in mice [188]. Nevertheless, the field of inflammatory Th1/Th17-mediated diseases that may benefit from such treatment is wide and studies of helminth-derived therapy have only just commenced. Much work remains to be done, including analyzing and extracting the molecules responsible for helminth regulating properties, clarifying their action on the immune system, confirming previous findings in larger prospective trials, and identifying other diseases eligible for this new type of immunomodulation. Furthermore, these immunotherapies require the greatest caution, especially regarding the unclear long-term effects of helminth immunomodulation. The manipulation of the immune response could lead to compromise in the defense mechanisms against other pathogens or cancers. The possibility of parasites inducing chronic infection, which may be less controllable, also needs to be considered. Therefore, the use of helminth-derived molecules appears to be a more attractive and safe solution [95,154]. If handled cautiously, helminthes might become part of our future therapeutic armamentarium.

## Summary

Since the first epidemiological observations that led to the birth of HH were reported, major advances have clarified both which microorganisms are involved in this protective cohabitation as well as their modulatory actions on immune cells, especially regarding helminthes’ effects. It is becoming apparent that these parasites are acting simultaneously at all levels and on the different key cellular players of the immune system establishing a real network aiming to promote a tolerant environment. Thus, helminthes hamper immune response to ensure their own survival and simultaneously protecting the host against the occurrence of chronic immune-mediated conditions by limiting the development of inflammation and autoimmunity. The promising results of the first clinical trials conducted in several autoimmune diseases using helminth-derived molecules provide an innovative therapeutic potential. However, many questions remain to be unraveled, including the type of helminthes that should be used, the type of diseases that should be targeted, and the long-term risks. Equally important is the elucidation of active molecules and the methods for their extraction. Answers for all the above need to be found before envisaging the widespread use of these novel immunotherapies.

## Abbreviations

Breg cells: B-regulatory cells
CD: Crohn’s disease
CDAI: Crohn’s disease activity index
CIA: Collagen-induced arthritis
CNS: Central nervous system
DC: Dendritic cell
DNBS: Dinitrobenzene sulfonic acid
DSS: Dextran-sodium-sulfate
EAE: Experimental autoimmune encephalitis
ES: Excretory-secretory
HH: Hygiene Hypothesis
IBD: Inflammatory bowel disease
IFN: Interferon
IL: Interleukin
ILC: Innate lymphoid cells
MS: Multiple sclerosis
NKT cells: Natural Killer T-cells
NOD: Non-obese diabetic
PC: Phosphorylcholine
PPR: Pattern recognition receptors
RA: Rheumatoid arthritis
SEA: Soluble egg antigen
SLE: Systemic lupus erythematosus
T1D: Type-1 diabetes
TGF: Transforming growth factor
Th cells: T-helper cells
TLR: Toll-like receptor
TNBS: Trinitrobenzene sulfonic acid
TNF: Tumor necrosis factor
Treg cells: T-regulatory cells
TSHR: Thyroid stimulating hormone receptor
TSO: *Trichuris suis* ova
UC: Ulcerative colitis
UCDAI: Ulcerative colitis disease activity index
